# Association of phase angle with sarcopenia in chronic musculoskeletal pain patients: a retrospective study

**DOI:** 10.1186/s13018-023-03567-1

**Published:** 2023-02-04

**Authors:** Hironori Tsuji, Tomoko Tetsunaga, Haruo Misawa, Keiichiro Nishida, Toshifumi Ozaki

**Affiliations:** 1 Department of Orthopedic Surgery, Okayama Red Cross Hospital, 2-1-1 Aoe, Kitaku, Okayama City, 700-8607 Japan; 2 Department of Orthopedic Surgery, Okayama University Hospital, 2-5-1, Shikata-Cho, Kitaku, Okayama City, 700-8558 Japan; 3 Department of Locomotive Pain Center, Okayama University Hospital, 2-5-1, Shikata-Cho, Kitaku, Okayama City, 700-8558 Japan; 4 Department of Orthopedic Surgery, Okayama University Graduate School of Medicine, Dentistry, and Pharmaceutical Sciences, 2-5-1 Shikata-Cho, Kitaku, Okayama City, 700-8558 Japan

**Keywords:** Sarcopenia, Chronic musculoskeletal pain, Phase angle, Bioimpedance analysis, Detection

## Abstract

**Background:**

In chronic musculoskeletal pain patients, detection of sarcopenia is of significant clinical interest. Phase angle, which can be measured through bioelectrical impedance analysis (BIA), can detect sarcopenia; however, the evidence in chronic musculoskeletal pain patients is limited. This study aimed to assess the relationship between phase angle and sarcopenia in patients with chronic musculoskeletal pain. Our hypothesis was that phase angle would be a useful indicator to identify sarcopenia in patients with chronic musculoskeletal pain.

**Methods:**

A total of 190 patients (51 men and 139 women) with chronic musculoskeletal pain were included in this retrospective cross-sectional study. Patient data of backgrounds, numeric rating scale score for pain, skeletal muscle index, and phase angle assessed using BIA were retrospectively reviewed. Sarcopenia was diagnosed using the Asian Working Group for Sarcopenia criteria 2019.

**Results:**

A total of 51 patients (26.7%), including 10 men (19.6%) and 41 women (29.5%), were diagnosed with sarcopenia. Phase angle, sarcopenia-related factors, age, and body mass index (BMI) differed significantly in patients with and without sarcopenia. On multiple logistic regression analysis, the prevalence of sarcopenia was significantly correlated with phase angle and BMI. The areas under the curve exhibited high accuracy in discriminating sarcopenia in men and moderate accuracy in both sexes and in women.

**Conclusions:**

Phase angle may be a valid discriminator of sarcopenia in patients with chronic musculoskeletal pain.

## Background

Chronic pain, which affects 20% of the general population, is a global problem that decreases activities of daily living [1, 2]. A total of 30–50% of older adults suffer from chronic pain, most of which originates from the musculoskeletal system [3]. Furthermore, musculoskeletal conditions are the leading cause of physical disability and also have a large impact on many other aspects of older people’s health, such as low physical activity level, poor mobility, frailty, depression, cognitive impairment, and falls [4]. As a musculoskeletal problem, sarcopenia, which was proposed to be a progressive and generalized loss of skeletal muscles, is attracting attention [5–9]. Sarcopenia is associated with increased adverse outcomes, including falls, fractures, functional decline, and even mortality [10–14]. In patients with chronic musculoskeletal pain, physical function and activity are impaired from an early age and exacerbate further with pain, which was explained by the fear-avoidance model [15–17]. This model explains why some patients with musculoskeletal disorders develop chronic pain syndrome. In this model, pain experience causes fear of pain itself, which leads to avoidance behavior and eventually immobilization and disuse syndrome, and disuse syndrome exacerbates pain. Therefore, it is very important to detect musculoskeletal dysfunction and perform physical exercise to maintain daily activity for the treatment of chronic musculoskeletal pain [18–20]. While chronic musculoskeletal pain and sarcopenia may correlate with each other, and detection of sarcopenia is important in the patients with chronic pain, the evidence for the association between them is limited.

On the other hand, phase angle, which can be measured noninvasively by bioelectrical impedance analysis (BIA), is reported to reflect the quality of cells, and a lower phase angle suggests decreased cellular integrity [21–23]. Previous reports have suggested that phase angle correlated with nutritional status, muscle strength, and mortality [24–26]. Therefore, phase angle could be used for sarcopenia detection [27–31]. However, in chronic musculoskeletal pain patients, it is unclear how phase angle and sarcopenia are related and whether phase angle can effectively detect sarcopenia. Therefore, the purpose of this study was to assess the relationship between phase angle and sarcopenia in patients with chronic musculoskeletal pain. Our hypothesis was that phase angle would be a useful indicator to identify sarcopenia in patients with chronic musculoskeletal pain.

## Methods

### Study participants

**Fig. 1:**
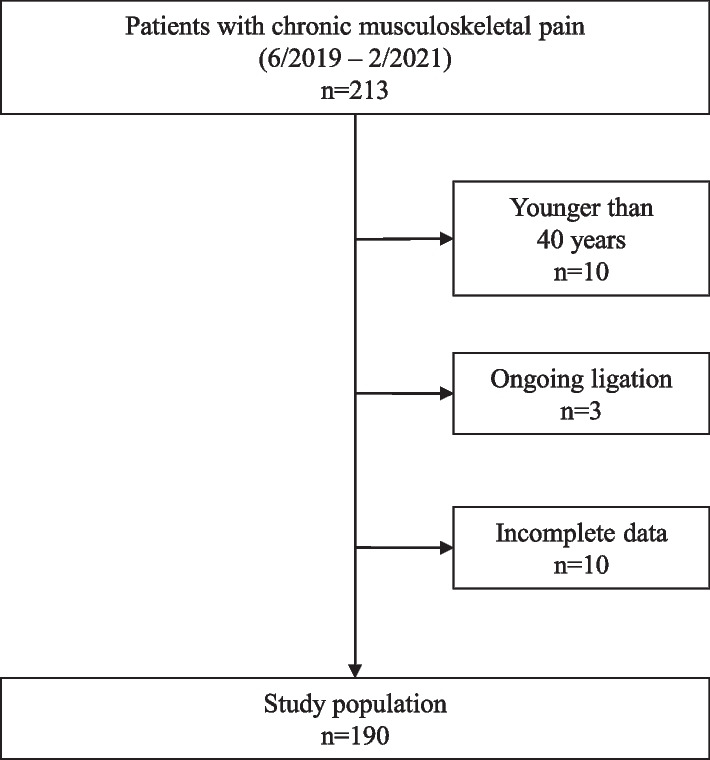
Flowchart showing study population and patient recruitment

This retrospective study was conducted at Okayama University Hospital. The participants included 190 patients (51 men, 139 women) with chronic musculoskeletal pain who visited our pain outpatient clinic between June 2019 and February 2021. The inclusion criteria for this study were age over 40 years, pain for longer than 3 months, and complete self-report questionnaires and physical examination. The exclusion criteria were as follows: in litigation, dementia, delirium, or other conditions that made completing questionnaires and physical examinations difficult (Fig. 1). Ethical approval was obtained from the hospital board of ethics, and the need for patient informed consent was waived due to the retrospective study design. This study was conducted in accordance with the Code of Ethics of the World Medical Association (Declaration of Helsinki) for experiments involving humans.

### Assessment of sarcopenia-related factors

#### Diagnosis of sarcopenia

Diagnosis of sarcopenia was performed according to the Asian Working Group for Sarcopenia (AWGS) criteria 2019 [32]. Gait speed, grip strength, and muscle mass were measured in this study. The criterion for low muscle strength was handgrip strength < 28 kg for men and < 18 kg for women and that for low physical performance was walking speed at < 1.0 m/s for 6 m. The skeletal muscle index (SMI) was assessed using InBody 770 and S10 (InBody Japan, Tokyo, Japan), and low muscle mass was defined by an SMI of < 7.0 kg/m^2^ in men and < 5.7 kg/m^2^ in women in this study. Sarcopenia was defined by the presence of low muscle mass combined with either low muscle strength or low physical performance.

### Assessment of phase angle

Phase angle was defined by the following equation using 50-kHz current:

Phase angle (degrees) = arctangent [reactance (Xc)/resistance (R)] × (180/*π*).

This calculation was performed automatically by the device, and we used the data obtained during SMI assessment. Diagnosis of sarcopenia and measurement of phase angle were performed by a same examiner.

### Evaluation of pain-related factors

#### Pain intensity assessment

The numeric rating scale (NRS) was used for assessment of pain intensity. NRS scores range from 0 to 10, with 0 representing no pain and 10 representing the worst imaginable pain [33]. The average pain intensity in the past 1 week was used in this study.

### Statistical analyses

Descriptive statistics are presented as mean ± standard deviations (SDs) for continuous variables and as numbers and percentages for categorical variables. The Kolmogorov–Smirnov test was used to assess normality for continuous variables. We analyzed correlations of phase angle with each measured variable using Spearman’s rank correlation coefficient. Then, we performed the Mann–Whitney U test to compare the measured parameters in the patients with and without sarcopenia. In a subsequent analysis, we performed multiple logistic regression analysis to evaluate the factors and odds ratio (OR) associated with sarcopenia. The explanatory variables included phase angle, body mass index (BMI), NRS, age, and sex. Next, in order to evaluate the discrimination performance of phase angle, area under the curve (AUC) was calculated using a receiver operating characteristic curve (ROC) analysis. Then, the sensitivity and specificity were calculated using the best cutoff point of phase angle for both genders and each gender individually with the Youden index for the ROC, respectively. As a previous study reported that men and women had different cutoff values for the phase angle, evaluation was conducted in both genders, male and female. The sample size was set as 10 of event per variable in logistic regression analysis [34]. Since this study was designed five variables in the multiple logistic regression analysis, the number of events, i.e., the required number of sarcopenia participants, was determined to be 50. We reviewed retrospectively all cases in the period that met the number of events. For the statistical analyses, we used EZR software (Saitama Medical Center Jichi Medical University, Tochigi, Japan), which is a graphical user interface for R (The R Foundation for Statistical Computing). Results were considered significant at a level of *p* < 0.05.

## Results

### Participants’ characteristics

The characteristics of the patients are shown in Table 1. The mean age was 67.2 (SD: 13.5) years, and the mean NRS score was 5.2 (SD: 2.5) points. Sarcopenia was diagnosed in 51 (26.7%) patients, which included 10 (19.6%) men and 41 (29.5%) women. The mean phase angle was 4.7 (SD: 1.0) degrees. Pain site and other sarcopenia-related factors are also shown in Table 1.

**Table 1:** Participant characteristics

Variables	All (*n* = 190)	Men (*n* = 51)	Women (*n* = 139)
Age (years)	67.2 ± 13.5	65.3 ± 12.2	67.9 ± 13.9
BMI (kg/m^2^)	23.6 ± 4.5	23.7 ± 3.7	23.5 ± 4.8
Sarcopenia	51 (26.7)	10 (19.6)	41 (29.5)
Grip power (kg)	19.6 ± 10.4	29.3 ± 12.0	16.1 ± 6.9
Gait speed (m/s)	1.1 ± 0.4	1.1 ± 0.4	1.3 ± 0.4
SMI (kg/m^2^)	6.7 ± 1.3	7.9 ± 1.1	6.1 ± 0.9
Phase angle (°)	4.6 ± 0.9	5.3 ± 0.9	4.4 ± 0.8
NRS (points)	5.2 ± 2.5	4.9 ± 2.8	5.3 ± 2.4
Pain site			
Cranio-cervical	32 (16.8)	11 (21.6)	21 (15.1)
Upper limb	41 (21.5)	15 (29.4)	26 (18.7)
Trunk	111 (58.1)	27 (52.9)	84 (60.4)
Lower limb	98 (51.3)	25 (49.0)	73 (52.5)

Data are expressed as mean ± standard deviation for continuous variables, and as percentages (%) for categorical variables. *BMI* body mass index; *SMI* skeletal mass index; *NRS* numeric rating scale

We performed the following three analyses: 1. correlations among phase angle and measured variable, 2. comparison between with and without sarcopenia patients, and 3. the discrimination capacity of phase angle for sarcopenia.

#### Correlations among phase angle and measured variable

Table 2 shows the correlation among phase angle and other variables. Phase angle was significantly correlated with age (*r* = − 0.54, *p* < 0.001), BMI (*r* = 0.21, *p* = 0.003), grip power (*r* = 0.61, *p* < 0.001), gait speed (*r* = 0.42, *p* < 0.001), SMI (*r* = 0.71, *p* < 0.001), and NRS (*r* = − 0.19, *p* = 0.007).

**Table 2:** Correlation between phase angle and measured parameters

Variables	Phase angle	
	r	*p*
Age	− 0.54	< 0.001
BMI	0.21	0.003
Grip power	0.61	< 0.001
Gait speed	0.42	< 0.001
SMI	0.71	< 0.001
NRS	− 0.19	0.01

*BMI* body mass index; *SMI* skeletal mass index; and *NRS* numeric rating scale

#### Comparison between with and without sarcopenia patients

Table 3 shows the characteristics of patients with and without sarcopenia. All sarcopenia-related factors, phase angle, age, and BMI differed significantly in patients depending on the presence of sarcopenia. In a multiple logistic regression analysis, sarcopenia prevalence was significantly correlated with phase angle (OR = 0.09, *p* < 0.001) and BMI (OR = 0.80, *p* < 0.001) (Table 4).

**Table 3:** Participant characteristics with and without sarcopenia

Variables	With sarcopenia (*n* = 51)	Without sarcopenia (*n* = 139)	*P-*value
Age (years)	74.3 ± 11.3	64.6 ± 13.4	< 0.001
Gender (men/women)	10 / 41	41 / 98	0.199
BMI (kg/m^2^)	21.2 ± 3.3	24.4 ± 4.6	< 0.001
Grip power (kg)	12.4 ± 5.4	22.3 ± 10.5	< 0.001
Gait speed (m/s)	0.9 ± 0.4	1.2 ± 0.4	< 0.001
SMI (kg/m^2^)	5.3 ± 0.5	7.1 ± 1.1	< 0.001
Phase angle (°)	3.9 ± 0.6	4.9 ± 0.8	< 0.001
NRS (points)	5.7 ± 2.3	5.0 ± 2.6	0.076

Data are expressed as mean ± standard deviation for continuous variables. *BMI* body mass index; *SMI* skeletal mass index; *NRS* numeric rating scale

**Table 4:** Multiple logistic regression analysis examining factors associated with sarcopenia

Variables	Odds Ratio	95% CI		*P*-value
		Lower	Upper	
Phase angle	0.09	0.03	0.23	< 0.001
BMI	0.80	0.71	0.91	< 0.001

*CI* confidence interval; *BMI* body mass index

#### The discrimination capacity of phase angle for sarcopenia

**Fig. 2:**
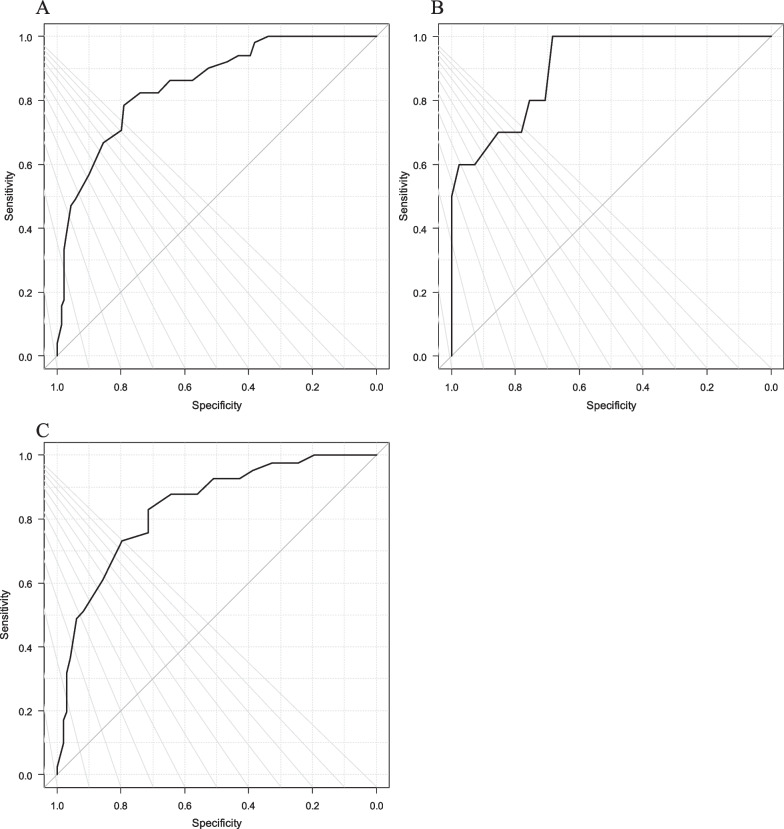
The receiver operating characteristic (ROC) curves of phase angle for discriminating sarcopenia based on gender: **A** both genders, **B** men, and **C** women

The discrimination value of phase angle for sarcopenia was assessed by the ROC curves (Fig. 2). The AUCs for both genders, men, and women were 0.851, 0.911, and 0.837, respectively. The cutoff values calculated by Youden index for both genders, men, and women were 4.2, 5.1, and 4.2, respectively (Table 5).

**Table 5:** AUCs and cutoff values of phase angle discriminating sarcopenia

	AUC	Cutoff value	Sensitivity (%)	Specificity (%)
All	0.85 (0.79–0.91)	4.2	78.4	79.1
Men	0.90 (0.81–1.00)	5.1	100	68.3
Women	0.84 (0.77–0.91)	4.2	82.9	71.4

*AUC* area under the curve. Values within parentheses show 95% confidence intervals. The sensitivity and specificity of the cutoff values are shown

## Discussion

Our findings showed that in chronic musculoskeletal pain patients, phase angle significantly correlated with age, BMI, grip power, gait speed, SMI, and NRS scores. Further, the phase angle was lower in patients with sarcopenia than in those without it. A lower phase angle was significantly correlated with sarcopenia and showed high accuracy in discriminating sarcopenia in men and moderate accuracy in both genders and women.

Previous reports suggested that phase angle was lower in individuals with sarcopenia, and several possible causes were reported to explain it. One of the most reported reasons was nutrition; patients with low phase angle were considered to be malnourished [27]. Previous studies that had taken BMI as a nutritional indicator reported that since patients with sarcopenia showed lower phase angle and BMI, they were considered to be malnourished [31]. In our study as well, with a multivariate analysis adjusted for age, gender, and degree of pain, a low phase angle and BMI were significantly correlated with sarcopenia prevalence. Given these, malnutrition could be factor in sarcopenia patients with chronic musculoskeletal pain, and therefore, nutrition therapy may be helpful in such patients. Though, in our study, nutritional status could only be assessed by BMI, further study would be needed to evaluate malnutrition in sarcopenia patients with chronic musculoskeletal pain. Another reason which should be considered is that phase angle correlates with functional status or muscle quality [28]. In our study, phase angle was significantly correlated with physical function and muscle strength, and we found that phase angle of patients with sarcopenia was lower than that of patients without it. Therefore, our results are compatible with those of previous reports.

As for the treatment of chronic musculoskeletal pain, our study suggests that detection and prevention of sarcopenia is of clinical significance in the management and treatment of musculoskeletal pain. Previous report suggested that phase angle was useful for detecting sarcopenia [27]. In addition, the AUC in ROC curves was 0.73 in kidney transplant recipients [31], 0.718 for men and 0.721 for women among community-dwelling individuals [28], and 0.85 in our study, which is similar to the results of previous studies. As for the cutoff point of phase angle, previous reports suggested that a value of 4.46 for kidney transplant recipients [31], 5.05 for cirrhosis patients [30], 4.55 for community-dwelling and hospitalized older adults [29], and 4.05 for men and 3.55 for women among community-dwelling individuals [28] could discriminate sarcopenia. Similarly, our results indicated that values of 4.2 for both genders, 5.1 for men, and 4.2 for women with chronic musculoskeletal pain were the best cutoff points to discriminate sarcopenia (Table 6). Thus, our study demonstrated that for chronic musculoskeletal pain patients, phase angle was useful in detecting sarcopenia.

**Table 6:** Phase angle for detecting sarcopenia in other studies

Authors (Year)	Country, sample size and age	Subjects	AUC of ROC curve	Phase angle cutoff points (degrees)
Kilic et al. (2017) [29]	Turkey,* n* = 263, M 110, W 153 > 65 yrs	Community-dwelling and hospitalized older adults	0.703	4.55
Yamada et al. (2018) [28]	Japan, *n* = 1009, M 285, W 724, M 81.1 ± 7.1 yrs, F 80.4 ± 6.8 yrs	Community-dwelling individuals	M 0.718 W 0.721	M 4.05 W 3.55
Espirito Santo Silva et al. (2019) [30]	Brazil, *n* = 119, M 54.4 ± 10.2 yrs	Cirrhosis patients	0.73	5.05
Kosoku et al. (2020) [31]	Japan, *n* = 210, M 122, W 88, median 55 range (45–66) yrs	Kidney transplant recipients	0.73	4.46
Our study	Japan, *n* = 190, M 51, W 139, 67.2 ± 13.5 yrs	Chronic musculoskeletal pain patients	All 0.85 M 0.90 W 0.84	All 4.2 M 5.1 W 4.2

*AUC* area under the curve; *ROC* receiver operating characteristic; *M* males; and *W* women

Notwithstanding the contribution of this study, there were several limitations. First, as this study was conducted in Japanese patients and we used the AWGS 2019 criteria for sarcopenia diagnosis, the results may differ in the studies involving other populations and those using other sarcopenia criteria, such as those proposed by the European working group for sarcopenia in older people [35] or the international working group on sarcopenia [36]. Second, it is known that psychosocial factors are intricately intertwined in patients with chronic musculoskeletal pain, and such factors were not considered in this study. Third, as this study was a retrospective cross-sectional study, it was difficult to evaluate the chronological order or change in phase angle, sarcopenia, and chronic musculoskeletal pain. Considering these limitations, future studies would require a detailed assessment, and clinical data from other countries would be needed to explore the relationships between phase angle, sarcopenia, and chronic musculoskeletal pain in different populations.

## Conclusion

Phase angle may be a valid discriminator of sarcopenia in patients with chronic musculoskeletal pain.

## Data Availability

The datasets generated during and/or analyzed during the current study are available from the corresponding author on reasonable request.

## Abbreviations

AUC: Area under the curve
AWGS: Asian Working Group for Sarcopenia
BIA: Bioelectrical impedance analysis
BMI: Body mass index
NRS: Numeric rating scale
OR: Odds ratio
ROC: Receiver operating characteristic
SD: Standard deviation
SMI: Skeletal muscle index
